# Integrating Neuromonitoring in Pediatric Emergency Medicine: Exploring Two Options for Point-of-Care Electroencephalogram (pocEEG) via Patient Monitors—A Technical Note

**DOI:** 10.3390/jpm13091411

**Published:** 2023-09-20

**Authors:** Leopold Simma, Fabrizio Romano, Steffen Schmidt, Georgia Ramantani, Bigna K. Bölsterli

**Affiliations:** 1 Emergency Department, University Children’s Hospital Zurich, University of Zurich, 8032 Zurich, Switzerland; 2 Children’s Research Center, University Children’s Hospital Zurich, University of Zurich, 8032 Zurich, Switzerland; 3 Division of Pediatric Emergency Medicine, Department of Pediatrics, Inselspital, Bern University Hospital, University of Bern, 3010 Bern, Switzerland; 4 Department of Neuropediatrics, University Children’s Hospital, University of Zurich, 8032 Zurich, Switzerland; 5 Child Development Center, University Children’s Hospital Zurich, University of Zurich, 8032 Zurich, Switzerland; 6 Department of Pediatric Neurology, Children’s Hospital of Eastern Switzerland, 9000 Sankt Gallen, Switzerland

**Keywords:** point-of-care EEG, simplified electroencephalogram, emergency department, non-convulsive status epilepticus, status epilepticus, altered mental status, pediatric emergency medicine, electroencephalogram, reduced lead electroencephalogram, rapid response EEG

## Abstract

Central nervous system (CNS) disorders are among the most frequent presentations in critically ill children. Status epilepticus (SE) is a frequent scenario in the resuscitation bay. In patients with altered mental status, non-convulsive SE (NCSE) is often underrecognized and critically impacts the neurological outcome and duration of hospitalization. An electroencephalogram (EEG) is required to diagnose NCSE. However, standard EEG recordings are time- and staff-intensive, and their availability is limited, especially outside regular working hours. We aimed to improve patient care by developing a simplified EEG recording method, using a reduced lead montage (point-of-care EEG—pocEEG), that is suitable for use in pediatric emergency departments. The objective was to devise a cost-effective unit with low space requirements that fitted the existing technical infrastructure. We present two technical options for clinical pocEEG acquisition using patient monitors (GE Carescape, Philips IntelliVue) that enable data collection for educational and research purposes. A simplified, rapid response EEG like the pocEEG enables neuromonitoring of patients with CNS disorders in pediatric emergency settings, facilitating timely diagnosis and treatment initiation when standard EEG is not readily available.

## 1. Introduction

Acute, nontraumatic disorders of the central nervous system (CNS) are among the most common emergencies [1] and rank among the chief presentations of critically ill children [2,3]. Changes in cortical electrical activity can be detected by electroencephalogram (EEG) recordings in various neurological emergencies, such as coma and acute encephalopathy. A condition of particular concern is status epilepticus (SE) [4,5,6,7,8]. The timely treatment of SE is critical as each minute of ongoing seizures increases the probability of an episode lasting more than 60 minutes and becoming super-refractory [9]. In encephalopathic patients, non-convulsive SE (NCSE) is an often underrecognized cause. Rapid diagnosis is vital for timely initiation of appropriate treatment, which in turn determines the outcome and duration of hospitalization [4,5]. Previous studies have shown that an EEG of encephalopathic patients can yield essential diagnostic information and substantially aid decision-making [10,11]. However, standard EEG recordings are time- and staffintensive, and their availability is limited, particularly outside regular working hours. Standard EEG machines use around 20 scalp electrodes to capture signals of the electrical brain activity. These signals are converted into visual representations on a screen for interpretation. Trained technicians place the electrodes in a standardized fashion (e.g., the 10–20 system) and neurophysiologists interpret the EEG signals. Consensus statements recommend neuromonitoring during critical care in all critically ill children and adults [12]. Under ideal conditions, neuromonitoring is performed in specialized centers via continuous or frequent intermittent EEG recordings, as well as through the interpretation of qualitative trends deriving from processed EEGs, such as the amplitude-integrated EEG: aEEG, in (predominantly neonatal) intensive care units [13]. However, long latencies to standard EEG initiation, even during regular working hours, have hindered its use for this indication, and have triggered the development of rapid emergent EEGs with reduced electrode montages [14]. Various commercial devices developed specifically for this purpose have been implemented and studied in adults [14], but none of them has been tested in children. Emergent and promising simplified EEG recording setups, such as the point-of-care EEG (pocEEG), may successfully bridge latencies to standard EEG initiation in the emergency department (ED). A simplified EEG performed at the bedside in the ED has been shown to complement clinical, laboratory, and imaging assessment and aid patient management [15,16]. A pocEEG has proven useful for evaluating altered mental status, detecting NCSE, and monitoring the treatment of ongoing SE [17]. In a retrospective pediatric cohort, 41/242 (17%) patients with altered mental status were diagnosed with non-convulsive seizures in pocEEG. Another retrospective study reported abnormalities in 17/86 (20%) children who underwent pocEEG in an ED setting [16]. These studies demonstrate that pocEEG may be beneficial to patient care when used by ED physicians [15]. However, all but one of these studies used dedicated EEG machines for pocEEG recording.

We aimed to develop a pocEEG recording unit suitable for use in a pediatric ED to improve timely detection of NCSE. We intended to devise a cost-effective unit with low space requirements, fitting the existing technical infrastructure for recording EEGs, and avoiding the acquisition of an entirely new device. Another objective was to enable EEG recording for review, educational, and study purposes. Here, we propose two solutions for pocEEG acquisition by combining a patient monitor with an EEG module.

## 2. Materials and Methods

### 2.1. Solution 1: GE Carescape

**Figure 1:**
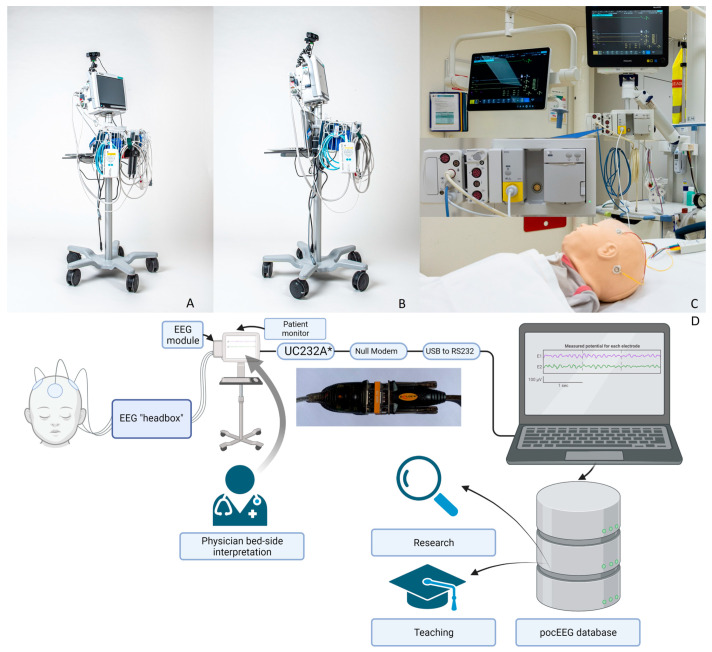
**Upper panel** (**A**–**C**) Technical equipment: first solution. Mobile point-of-care EEG (pocEEG) module, constructed using a mobile GE Carescape patient monitor, and a laptop computer mounted to the stand, shown here in frontal (**A**) and lateral view (**B**). Video camera on flexible tripod attached to monitor top. (**C**) Technical equipment: second solution. EEG module installed in the resuscitation bay, with a fixed Philips IntelliVue patient monitor. **Lower panel** (**D**): system diagram pocEEG recording; * ATEN USB to serial adapter (USB to RS-232 Adapter, UC232A, ATEN, Taiwan) is used for the GE Carescape system. Philips IntelliVue connection shown in Figure A1.

The GE Carescape series (GE Healthcare, Helsinki, Finland) are a set of standardized vital sign monitors with inputs including peripheral oxygen saturation, ECG, non-invasive blood pressure and outputs including peripheral oxygen saturation, heart rate, ECG, respiratory rate, arterial blood pressure. It can be fitted with an additional EEG module that captures recordings from up to four channels. For this project, we adapted and improved a previously published setup [17] to enable EEG and video recording. The standard mobile patient monitor (Carescape B450, GE Healthcare, Helsinki, Finland) available at the institution was configured for use with the EEG module as a standalone. Figure 1 illustrates the whole setup. To maximize flexibility, we adapted a mobile monitor for integration with our EEG module to allow its use in various ED bays (Figure 1A,B).

#### 2.1.1. EEG Module 

The EEG module set consists of the module itself and is connected to a “headbox” via cable. The module is inserted into a slot on the side of the patient monitor. EEG electrodes are connected to the headbox. The electrode positions for this setup are F7/F8 (antero-temporal region) and T5/T6 (retro-auricular/postero-temporal region), placed according to the international 10–20 system, with an additional reference electrode placed in the middle of the forehead. This results in two EEG channels, which are shown on the patient monitor along with the oxygen saturation and ECG signals. The EEG signal voltage is preset to +/−100 µV by the monitor, but the scale can be adjusted up to +/−500 µV on the monitor’s touchscreen. The technical specifications are shown in the Appendix A Table A1. We primarily used self-adhesive neonatal ECG-Electrodes (Kendall^®^ ECG electrodes; CardinalHealth, Dublin, Ireland, see also Section 2.3). These electrodes are the standard for ECG monitoring at this site, providing logistic advantages.

#### 2.1.2. Data Capture

The monitor does not have dedicated storage media as it was originally intended only for displaying signals. Therefore, a workaround is required to save the pocEEG signals. Monitor signals can be recorded via a laptop computer through a USB cable. GE Carescape monitors require an ATEN USB to serial adapter (USB to RS-232 Adapter, UC232A, ATEN, New Taipei, Taiwan) for data recording. We connected a standard notebook (HP ProBook 450 G3, Hewlett-Packard, Palo Alto, CA, USA) to a Delock USB to RS-232 (Delock, Berlin, Germany) adapter and used a null-modem adapter to connect both cables. We used the open-source VitalRecorder software (version 1.8.16.4) for signal recording [18]; it is freely available for download (http://www.vitaldb.net, first accessed on 12 March 2021). This software can record the vital signs of various patient monitors and anesthesia devices. Initial recordings are displayed as +/−100 µV. Clinical events can be logged in the software with a time stamp for medication administration, manipulation, and so forth. Once saved, the data tracks can be displayed at appropriate amplitude and voltage for review and analysis. Data may be exported to various file formats for further processing and analysis. A comprehensive manual is available online at vitaldb.net (http://www.vitaldb.net, accessed on 12 March 2021), which includes instructions on connection to various medical devices.

To ensure correct cable connection and thus pocEEG recording at all times, we mounted the notebook on the monitor stand (Figure 1B).

#### 2.1.3. Video pocEEG

For post hoc EEG analysis, simultaneous video recordings are acquired to facilitate the retrospective interpretation of clinical presentation, particularly of seizure semiology, and of EEG recordings, particularly of artifacts (Figure 1A,B).

An option for low-cost video capture is the Logitech C922 Pro Stream webcam (Logitech Europe S.A., Lausanne, Switzerland). This camera is compatible with the Logitech Capture (Logitech Europe S.A., Lausanne, Switzerland) software (version 2.08.11), which is freely available from the manufacturer’s website. The software can combine various image sources into a single video and combine the clinical webcam video and VitalRecorder EEG screen (Appendix A Figure A2) on a laptop computer. Depending on the resolution of the recorded video, one hour of recording is expected to result in a video file of 1–2 gigabytes in size. Video recording could also be accomplished with other camera and software solutions. Currently, only the GE system’s setup has been used with simultaneous video recording.

### 2.2. Solution 2: Philips IntelliVue

The Philips IntelliVue series are a set of standardized vital sign monitors with inputs, including peripheral oxygen saturation, ECG, non-invasive blood pressure and outputs including peripheral oxygen saturation, heart rate, ECG, respiratory rate, arterial blood pressure. A Philips IntelliVue Monitor MX700 (Philips Medical Systems, Boeblingen, Germany) with the Philips M1027B EEG module (Philips Medical Systems, Boeblingen, Germany) was used for this setup. A previously described pocEEG used the Philips IntelliVue Monitor MX550 [17].

#### 2.2.1. EEG Module

**Figure 2:**
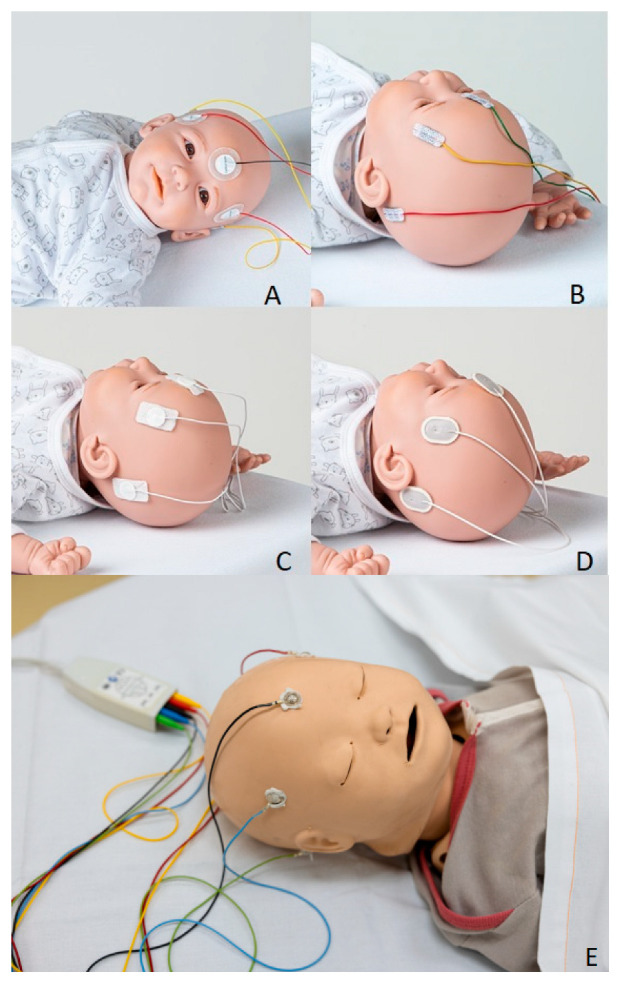
Electrode placement for point-of-care EEG (pocEEG) recordings; (**A**) CardinalHealth electrodes; (**B**) Micro Neolead^®^ electrocardiogram (ECG) electrodes; (**C**) Natus electrodes; (**D**) Ambu Neuroline 720^®^ electrodes; (**E**) Philips Cup electrodes; The electrodes here have been placed on the scalp, corresponding to the positions, F7/8, T5/6, of the international 10–20 system.

This module is also inserted into a monitor slot. The electrodes are connected to the module by an EEG cable. (Figure 1C) The monitor can adjust voltage up to 1000 µV; the standard preset is also 100 µV. ECG signals and saturation are shown on the same screen when connected (see Appendix A Figure A3). This system is implemented with cup electrodes and uses the electrode positions, F7/F8 and T5/T6, with a reference electrode placed in the middle of the forehead (see Section 2.3 and Figure 2E).

#### 2.2.2. Data Capture

**Figure A1:**
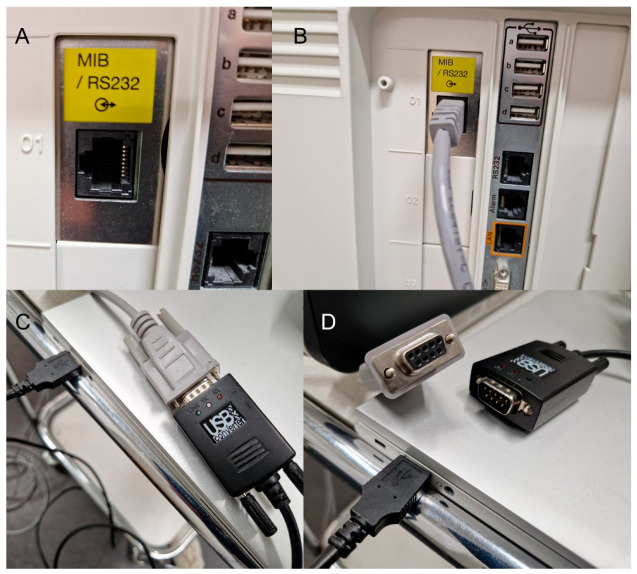
Data storage Philips IntelliVue system MX700. (**A**) Data port MIB; (**B**) Dataport with network cable (RJ45 to serial); (**C**,**D**) RJ45 to RS232 (grey, female) connector and USB to RS232 connector (black, male).

**Figure A2:**
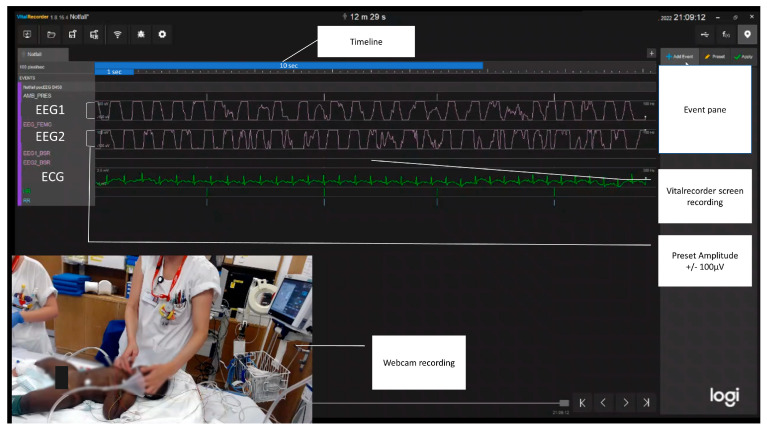
Screenshot of video pocEEG showing a non-convulsive status epilepticus in an 11-month-old infant; Video pocEEG: simultaneous video recording with Logitech Capture^®^ of the Logitech camera and laptop screen recording of the VitalRecorder software; channels: EEG 1 F7/T5 and EEG 2 F8/T6.

Additionally, Philips IntelliVue monitors offer no storage function to enable retrospective EEG analysis, as it is only intended for displaying signals. High-resolution data recording from Philips devices is possible with the ixTrend Expess software application (version 2.1.0.F.w14; Ixitos, Berlin, Germany), which is a commercial solution. An IntelliVue connector cable using a network connector (RJ45) to serial (RS232, female) and USB to serial (USB-RS232; male) cable is needed for data transmission to a computer notebook (see Appendix A Figure A1). Saved data tracks can be displayed at appropriate amplitude and voltage for review and analysis.

### 2.3. Electrodes

We used several different self-adhesive hydrogel scalp electrodes to capture pocEEG. In line with previous reports [17], we used standard neonatal Kendall^®^ ECG electrodes (CardinalHealth, Dublin, Ireland), which are readily available in the ED (GE Carescape site). We compared these with “small-footprint” Micro Neolead^®^ ECG Electrodes (Neotech Products, Valencia, CA, USA), “medium-footprint” OBM Neonatal Hydrogel Sensors^®^ (Natus Medical Inc., Middleton, WI, USA), Ambu^®^ WhiteSensor 40556 (Ambu A/S, Ballerup, Denmark) and “large-footprint” neurology surface electrodes Ambu Neuroline 720^®^ (Ambu A/S, Ballerup, Denmark) (see Figure 2A–D and Table 1).

Skin is often prepped with Cavilon (3M, St. Paul, MN, USA) to enhance attachment, especially in febrile patients. Cavilon is a skin barrier film that we routinely use in the pediatric ED to enhance adhesion of dressings of intravenous lines to moist, sweaty skin.

Neonatal and pediatric ECG electrodes (e.g., Hydrogel ECG Electrodes, AMBU A/S, Ballerup, Denmark) have been used for EEG recordings with the Philips monitor [17]. Reusable EEG cup electrodes, commonly used for standard EEG recordings, can be also used for pocEEG recordings (Philips—M1931A reusable adult cup electrode, Philips Medical Systems, Boeblingen, Germany). Cup electrodes require more skin prepping (OneStep Abrasiv Plus, Duelmen, Germany) and the application of electrode cream (SAC2 Electrode Cream, Spes medica, Newburyport, MA, USA).

## 3. Results

**Figure 3:**
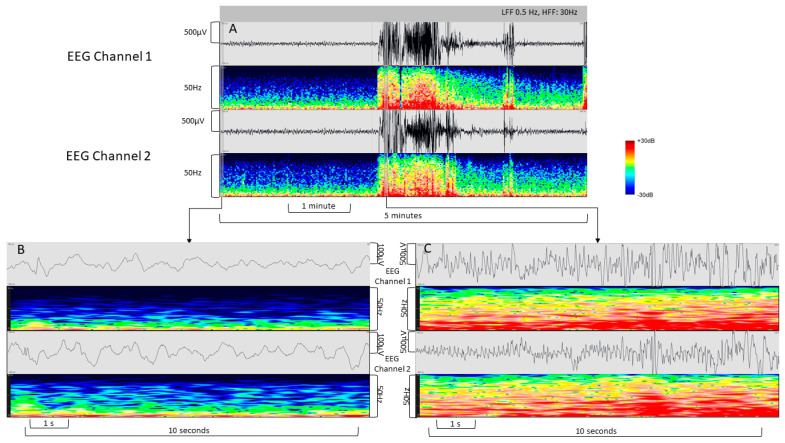
Raw EEG and colorcoded spectral array of the two pocEEG channels (F7/8, T5/6) from the GE System recorded with VitalRecorder. (**A**) Five-minute recording with a self-limiting seizure. (**B**) Raw EEG and color-coded spectral array prior to the seizure. (**C**) Bilateral seizure activity. LFF = low frequency filter; HFF = high frequency filter (HFF).

**Figure 4:**
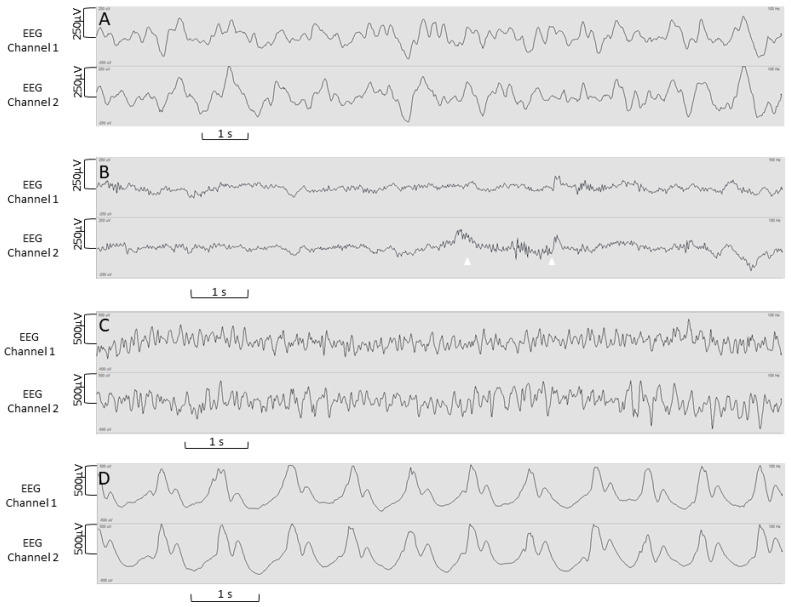
pocEEG recordings via VitalRecorder (mobile GE Carescape option); 14-month-old infant, recorded during sleep, amplitude 250 µV (**A**) delta-theta activity; (**B**) beta activity (arrowheads); (**C**) 4-month-old infant, amplitude 500 µV, bilateral discharges during a self-limiting seizure; (**D**) 16-month-old infant, amplitude 500 µV, bilateral discharges, febrile non-convulsive status epilepticus; Low frequency filter (LFF) 0.5 Hz, High frequency filter (HFF): 30 Hz.

During the implementation phase of the GE system, 62 pocEEGs were recorded within the scope of a quality improvement project. Out of these 62 pocEEGs, 21 (34%) were performed in females and 29 (47%) of the patients had a preexisting comorbidity (either neurologic or affecting other organ systems). The median age was 40.5 months (Min.–Max. 0–207 months, IQR 11.0–83.3 months). We present example tracings of patients who underwent pocEEG in the pediatric ED (Figure 3 and Figure 4).

The pocEEGs were used for neuromonitoring in patients with suspicion for seizure activity and were interpreted by pediatric emergency physicians on the monitor screen at the bedside. Traces were used clinically to guide further treatment. Fundamental EEG features are readily identifiable. These include predominant background activity, symmetry, sporadic epileptic discharges, and electrographic/electroclinical seizures (Figure 3 and Figure 4). Examples of seizure activity on pocEEG traces are available as Supplementary Material (Videos S1 and S2); both video examples show temporal and spatial evolution of the seizures. For most cases, the recording duration ranged from very brief (5 min) to longer monitoring periods (60–90 min) depending on the clinical scenario. We obtained EEG recordings using self-adhesive ECG electrodes (60/62 Kendall ECG, and 2/62 Ambu Neuroline).

**Figure 5:**
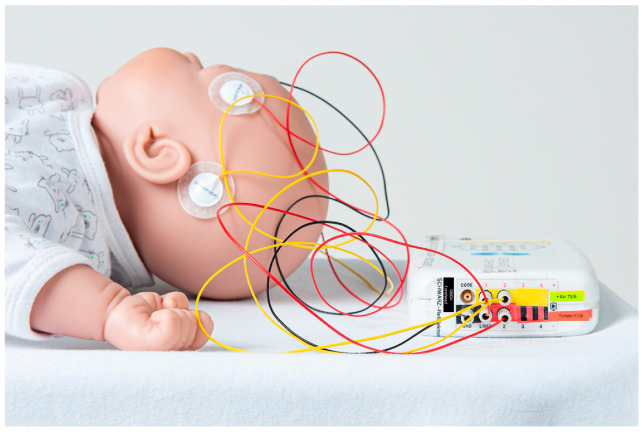
Visual cognitive aid on headbox for neonatal ECG electrodes (color code—yellow, ear T5/6; red, temple F7/F8; black, forehead; GE EEG module (“headbox”); Kendall neonatal ECG electrodes).

When reviewed by expert neurophysiologists, the electrodes mentioned in Table 1 yielded signals that were deemed to be of sufficient quality to be readily interpretable. After prepping the skin with Cavilon, impedances were below 10 kΩ when using these electrodes (Table 1). However, one electrode set (Micro Neolead^®^) still had impedances above the recommended level. The Kendall^®^ ECG electrodes are the standard ECG-monitoring electrodes at the site using the GE monitor. They are cheaper (Table 1), and staff are familiar with the product. Another advantage of using these ECG electrodes is their color-coded appearance, which provides a visual cue when connecting the electrodes to the head box (Figure 5). Importantly, in this technical note, we did not aim to validate pocEEG, which would require a simultaneous recording of standard EEG.

**Figure 6:**
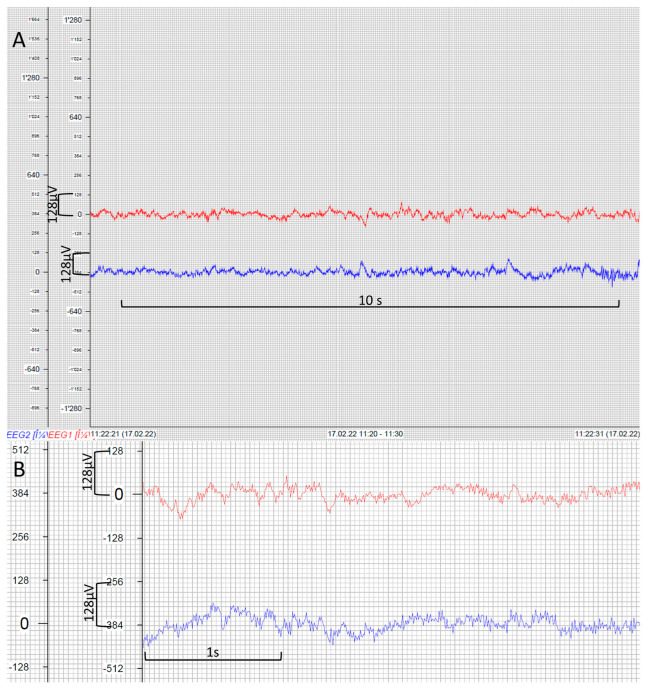
Screenshot of ixTrend Express point-of-care EEG (pocEEG) recording (Philips IntelliVue system) EEG channel 1 (red), EEG Channel 2 (blue); x-axis EEG amplitude in µV; (**A**) normal activity, 10 s segment; (**B**) close-up of EEG trace.

**Figure A3:**
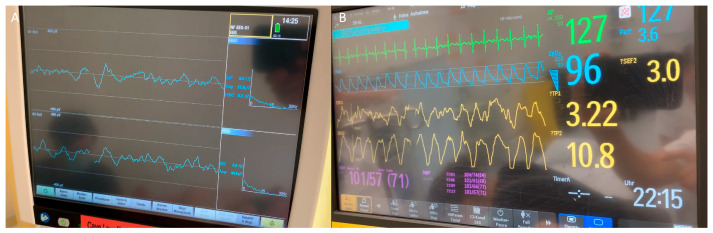
pocEEG tracings on a GE B450 Carescape monitor, normal activity (**A**) and on a Philips IntelliVue MX550 with seizure activity (**B**).

The site that uses the Philips IntelliVue system evaluates about 30 patients per year with altered mental status and suspected NCSE. These are also reviewed by the local neurophysiologists. A recording of the Philips IntelliVue system with reusable cup electrodes via the IxTrend Express software (Ixitos, Berlin, Germany) is shown in Figure 6. Images during clinical neuromonitoring on monitor systems of both manufacturers are shown as Supplementary Material (Appendix A Figure A3).

## 4. Discussion

Previous studies investigating the application of simplified EEG in the pediatric ED have used dedicated EEG recording devices [15,16]. Although a dedicated EEG device with simultaneous video and EEG monitoring may be considered, the current starting price of USD 16,000 would be prohibitive for many pediatric emergency departments. The current starting price of USD 4000 for an EEG module would potentially be much more affordable. Apart from the price, space constraints may have to be considered before introducing an additional device to the device-ridden ED setting. Introduction and staff familiarization with a new device that may not be used daily may also be an obstacle. For all these reasons, upgrading familiar ED devices with an additional EEG module could be a more straightforward and more feasible option and suitable for both high- and low-resource settings.

The application of pocEEG in the ED should be kept as simple as possible, including electrode placement. For this reason, the recording electrodes are placed below the hairline. “Subhairline” EEG is a viable option for EEG monitoring in the adult intensive care unit [19]. Another consideration is training, as the EEG electrodes will be placed by ED nurses and physicians that are not familiar with EEG. We used a two-channel EEG approach, including the electrode positions, F7/F8 and T5/T6 (retroauricular/posterotemporal region), according to the 10–20 system, as previously published [17]. The precise positioning of the recording electrodes in the 10–20 system was not explicitly specified in a previous study focusing on two-channel EEG recordings in the ED; that study reported bilateral frontopolar and parietal electrodes with two reference electrodes [16], but the electrode positions were likely Fp1/2 and P3/P4 (imprint on EEG examples in the paper). In another study on simplified EEG, four channels over the frontal (Fp1-A1 and Fp2-A2) and occipital regions (O1-A1 and O2-A2) were used along with reference electrodes, resulting in nine electrodes [15]. More channels will sample EEG signals from a more extensive cortical region and may thus be more sensitive, as has been demonstrated for subhairline EEG compared to standard EEG [19]. However, more electrodes will inevitably increase the complexity of electrode placement for non-expert staff.

### 4.1. Limitations

The deployment of an EEG module in an ED environment comes with various pitfalls. Depending on the manufacturer’s presets or installed software versions, the EEG module may not work immediately with a given patient monitor. This pitfall can be avoided by carefully planning the groundwork with the local medical engineering department and the manufacturers’ representatives. This factor may also influence the decision if a department chooses to use pocEEG in a dedicated area like a resuscitation bay (Philips IntelliVue system) or to opt for a mobile solution (GE Carescape system). Nevertheless, the local floor plan may dictate where such a system is best implemented. Storing the pocEEG traces may be accomplished via the software mentioned in the Section 2. However, the recording may fail due to operator errors and technical problems such as low laptop batteries. This can be overcome with repeated training and simple, well-written instructions attached to the devices. Additionally, attaching data and power cables permanently to the monitors and the laptop computers minimizes these errors. For example, in the GE Carescape system, the laptop computer is fixed to the mobile monitor (Figure 1A,B). The power cables for the mobile monitor and the laptop computer are in a multi-plug connector to prevent their batteries from running out of power.

Video recording requires significantly higher computing power and data storage than EEG recording alone with the frugal VitalRecorder software. After a mandatory institutional hardware upgrade from an HP ProBook 450 G3 (Hewlett-Packard, Palo Alto, CA, USA) to a newer HP EliteBook 850 G8 (Hewlett-Packard, Palo Alto, CA, USA), we noticed improved video resolution due to more computing power. However, such changes in components also carry risks and require careful retesting. Some of these mentioned challenges of good quality physiologic data collection in the emergency department have been reported by other authors [20]. Of note, EEG recordings obtained via patient monitors cannot meet the same quality standards as a conventional EEG applied by skilled neurophysiology technicians. While the technical capabilities of monitor modules are clearly less advanced (Appendix A Table A1), acceptable impedances for continuous EEG below the recommended 10 kΩ threshold are achievable, also with ECG electrodes [21,22]. We found that using ECG electrodes offers practical advantages, as they are cheaper than EEG electrodes, familiar to ED staff, and feature colored cables and plugs, which is especially helpful in a busy emergency department setting. These factors were the main reasons for the electrode selection at the site using the GE system. However, cup electrodes for EEG recording are also available with colored cables (Figure 2E). This type is in use at the site using the Philips system, but cup electrode placement with skin preparation may take longer and requires more training. The provided information may help departments decide which components of the setups to implement in their respective setting. In our opinion, good cooperation between pediatric emergency medicine and pediatric neurology is essential for the introduction of pocEEG. The use of this technique must be endorsed by both departments to ensure its successful implementation.

### 4.2. Remote EEG Interpretation

While there is evidence that individuals without expertise in EEG can acquire basic EEG interpretation skills [23], remote EEG interpretation by the neurologist on-call may be desirable depending on local circumstances. Previous studies have utilized instant messengers for expert input [17], but this approach may not align with current data protection policies in hospitals. VitalRecorder is able to provide web-based remote monitoring (www.vitaldb.net, accessed on 12 March 2021), but it is not intended for clinical use [18]. Another alternative for remote EEG interpretation could be utilizing a web-conferencing tool already in place at the hospital. However, ensuring secure access for remote data review may pose challenges.

### 4.3. Outlook

A recent review on reduced montage, rapid response EEG approaches, which includes the precursor of the presented set up, concluded that these technologies are promising and can lead to a cost-effective solutions for neuromonitoring with reduced dependence on expert staff [14]. Nonetheless, there are still several research questions that need to be addressed through future studies. These include optimal electrode positioning for pocEEG recording and the pocEEG sensitivity. Furthermore, adequate training and education will be fundamental for the successful implementation and use of this promising technique. Thus, additional research is needed to determine the best training modalities for pediatric emergency medicine providers. However, reports from other critical care settings have shown that teaching basic EEG skills to EEG non-experts is a feasible option [23]. The implementation of pocEEG may be tailored, depending on needs and the local infrastructure. A pocEEG is immediately available on patient arrival, whereas few institutions have access to 24/7 standard EEG in the ED. The EEG traces permit interpretation of cortical activity at the bedside and may guide treatment or interventions. Additionally, pocEEG may also be used to bridge time or to decide on the urgency of a standard EEG until the neurophysiology team arrives at the bedside.

## 5. Conclusions

This work illustrates our approach for establishing simplified, two-channel pocEEG units in the pediatric ED, using patient monitors from two manufacturers for integration with the EEG recording module. Neuromonitoring is key to make the diagnosis of NCSE and may help to optimize management of convulsive SE. Our pocEEG setups enable the prompt initiation of neuromonitoring in the ED when required and may thus help to close a gap in patient care in settings where standard EEG is unavailable or until standard EEG has been mounted.

## Figures and Tables

**Table 1:** Overview Electrode Types for point-of-care EEG.

Product	Shape/Cables	Size	Price Per Pack	Electrode Impedance (kΩ)
*Self-adhesive electrodes—“small footprint”*				
Micro Neolead^®^ ECG Electrodes (Neotech Products, Valencia, CA, USA)	Rectangular, colored cable	9 mm × 14 mm w/adhesive 9 × 25 mm	8.55 USD (Pack of 3) (17.10 USD per patient)	12–16 kΩ
*Self-adhesive electrodes—“medium footprint”*				
Neonatal Kendall^®^ ECG electrodes (CardinalHealth, Dublin, Ireland)	Round, colored cable	Diameter 17 mm, w/adhesive border 30 mm	1.25 USD (Pack of 3) (2.50 USD per patient)	4–6 kΩ
OBM Neonatal Hydrogel Sensors^®^ (Natus Medical Inc., Middleton, WI, USA)	Rectangular, white cable	Diameter 10 mm w/adhesive 30 mm × 18 mm	4.10 USD (Pack of 5) (4.10 USD per patient)	N/A
Ambu^®^ White Sensor 40556 (Ambu A/S, Ballerup, Denmark)	Square, colored cable	Diameter 17 mm, w/adhesive border 22 mm × 22 mm	2.75 USD (Pack of 3) (5.5 USD per patient)	7–8.5 kΩ
*Self-adhesive electrodes—“large footprint”*				
Ambu Neuroline 720^®^ (Ambu A/S, Ballerup, Denmark)	Rectangular (rounded), white cable	30 mm × 21 mm	22 USD (Pack of 12) (22 USD per patient)	5–6 kΩ
*Reusable electrodes*				
Philips M1931A Reusable EEG Adult Cup Electrode (Philips Medical Systems, Boeblingen, Germany)	Round, colored cable	Diameter 10 mm, silver	110 USD (Pack of 6)	<5 kΩ

**Figure A4:**
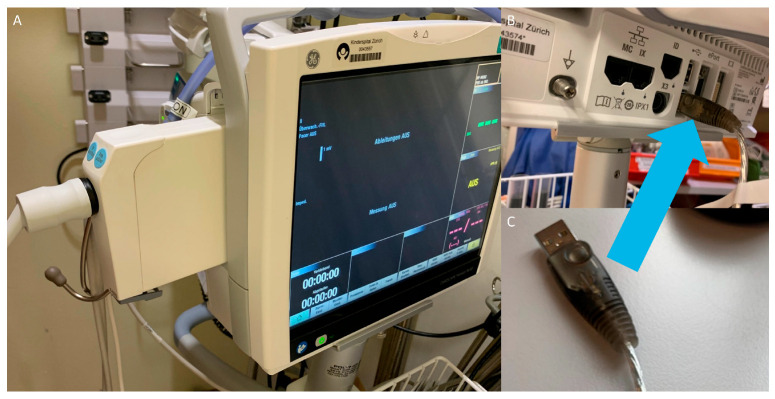
EEG module in side port of GE Carescape B450 (**A**), USB connection ports (blue arrow) at the rear of the GE Carescape B450 for data recording (**B**); USB cable for data recording, (USB to RS-232 Adapter, UC232A, ATEN, Taiwan; (**C**)).

## Data Availability

All data are published in this article.

## Supplementary Materials

The following supporting information can be downloaded at https://www.mdpi.com/article/10.3390/jpm13091411/s1, Video S1: Evolution of a generalized epileptic seizure on pocEEG from Figure 4C, 4-month-old infant; Video S2: Evolution of a generalized epileptic seizure on pocEEG, 3-month-old infant.

## Appendix A

Appendix A includes images of technical details of the device setup, connection of devices, and views of the screens during pocEEG recording to facilitate replication in other settings (Figure A1, Figure A2, Figure A3 and Figure A4). Table A1 lists the technical specifications of the GE EEG module and the Philips IntelliVue EEG module.

**Table A1:** Technical specifications EEG modules.

	GE Carescape EEG Module	Philips IntelliVue EEG Module
Sampling rate	100 Hz per channel	125 Hz per channel
Range	±400 µV	±1000 µV
Input filter	0.5 to 30 Hz	0.5 to 30 Hz
Input impedance	>8 MΩ at 10 Hz	>15 MΩ at 10 Hz
Impedance limit	5 (10) kΩ	5 kΩ (up to 30 kΩ)
